# Synthesis, crystal structure and Hirshfeld surface analysis of bis­(caffeinium) hexa­chlorido­platinum(IV) in comparison with some related compounds

**DOI:** 10.1107/S2056989023005157

**Published:** 2023-06-16

**Authors:** Karim A. Zagidullin, Anton P. Novikov, Daria A. Zelenina, Mikhail S. Grigoriev, Konstantin E. German

**Affiliations:** a Frumkin Institute of Physical Chemistry and Electrochemistry Russian, Academy, of Sciences, 31 Leninsky Prospekt bldg 4, 119071 Moscow, Russian Federation; b Peoples’ Friendship University of Russia (RUDN University), 6 Miklukho-Maklaya, St, 117198, Moscow, Russian Federation; c Kyrgyz-Russian Slavic University, 6 Chuy Avenue, Bishkek, Kyrgyzstan; University of Durham, United Kingdom

**Keywords:** crystal structure, platinum, Pt, caffeine, Hirshfeld surface analysis, hexa­halide, π-stacking

## Abstract

The mol­ecular and crystal structure of the title compound, (C_8_H_11_N_4_O_2_)_2_[PtCl_6_], synthesized from hexa­chloro­platinic acid and caffeine in methanol, was studied by single-crystal X-ray diffraction. The caffeinium cations form a double layer *via* hydrogen bonds and π-stacking inter­actions. The Hirshfeld surface analysis showed that the largest contribution to the crystal packing is made by H⋯H (31.2%), H⋯Cl/Cl⋯H (22.6%), O⋯H/H⋯O (21.9%) contacts for the cation and H⋯Cl/Cl⋯H (79.3%) contacts for the anion.

## Abstract 2

In the crystal, the cations and anions form a layered structure *via* strong N—H⋯Cl, weak C—H⋯Cl and C—H⋯O hydrogen bonds and π-stacking inter­actions.

## 1. Chemical context

Caffeine is a biologically active compound involved in a number of biochemical processes (Costa *et al.*, 2010 ▸; Santos *et al.*, 2010 ▸; Herman & Herman, 2012 ▸). Some sources consider it the most common medicine in the world, constantly used by the population (Knapik *et al.*, 2022 ▸). It is known that caffeine compounds are able to exert a strong influence on the action of various pharmaceutical drugs (Traganos *et al.*, 1991 ▸). Currently, an active search is underway for platinum-based pharmaceutical drugs, primarily those with anti­tumor activity (Dilruba & Kalayda, 2016 ▸). In this regard, it seemed important to us to study the inter­action of caffeine with the chemical forms of platinum used in the pharmaceutical industry. In addition, platinum is actively used as a catalyst in chemical reactions, including various fields of fine organic synthesis (Blaser & Studer, 2007 ▸; Zhang *et al.*, 2006 ▸; Seselj *et al.*, 2015 ▸). Study of inter­action of Pt^IV^ with various heterocyclic organic mol­ecules is of great importance in the context of search for new catalytic reactions and synthetic routes. Studies on the inter­action of hexa­chloro­platinates with various biological organic compounds have been performed before, for example by Novikov *et al.* (2021 ▸, 2022 ▸).

In this work, the title compound **I** containing [PtCl_6_]^2−^ anions and caffeinium cations was synthesized by the reaction of caffeine with H_2_[PtCl_6_] in methanol and structurally characterized, using Hirshfeld surface analysis to estimate relative contribution of non-covalent inter­molecular inter­actions in comparison with similar compounds, bis­(3-carb­oxy­pyrid­in­ium) hexa­chloro­platinum RECJAO (**II**; Novikov *et al.*, 2022 ▸) and methyl­caffeinium hexa­fluoro­phospate AXUQIT (**III**; Kascatan-Nebioglu *et al.*, 2004 ▸). 
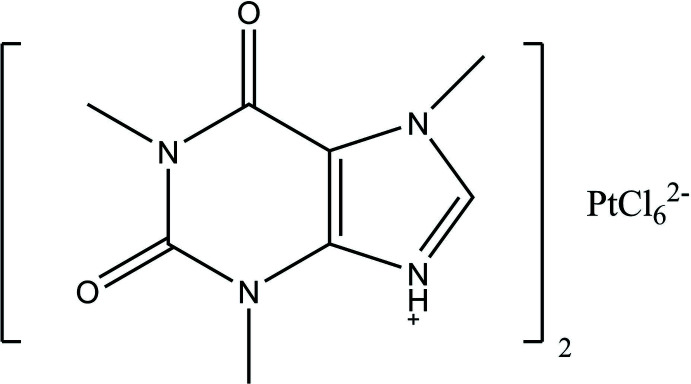


## 2. Structural commentary

**Figure 1:**
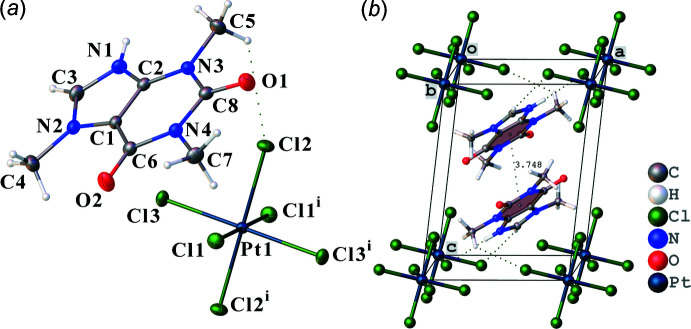
(*a*) Mol­ecular structure of the title compound and (*b*) the unit cell with π-stacking inter­actions [symmetry code: (i) −*x*, −*y*, −*z*].

Compound **I** (Fig.1*a*) crystallizes in the triclinic space group *P* 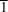 . The unit cell (Fig. 1 ▸ *b*) contains two caffeinium cations and one centrosymmetric hexa­chloro­platinate anion with a platinum atom in a special position 1*a*. In the imidazole ring of the caffeine mol­ecule, the nitro­gen N1 atom is protonated. The cation, including the methyl groups, has a flat geometry (maximum deviation for non-hydrogen atoms 0.030 Å). The [PtCl_6_]^2−^ anion has a slightly distorted octa­hedral geometry with similar Pt—Cl bond distances (Table 1 ▸).

## 3. Supra­molecular features

Hydrogen bonds and π-stacking play a significant role in the formation of inter­molecular inter­actions in the crystal structure of **I**. π-stacking is observed between the six-membered pyrimidine rings. Pairs of parallel cations related by an inversion centre, are stacked with inter­planar separation of 3.404 (3) Å (Fig. 1 ▸ *b*).

Similarly, π–halogen inter­actions (Lucas *et al.*, 2016 ▸; Savastano *et al.*, 2018 ▸; Frontera *et al.*, 2011 ▸; Novikov *et al.*, 2022 ▸) are found between the aromatic ring C6/N4/C8/N3/C2/C1 (centroid *Cz*) and chlorine atoms Cl1 and Cl2, with *Cz* ⋯ Cl distances of 3.8643 (11) and 3.7170 (11) Å, respectively, and α angles between the ring plane and the *Cz*⋯Cl vector of 61.82 (7) and 62.28 (7)°, respectively. It is uncertain whether such an inter­action exists with Cl3 [*Cz*⋯Cl = 4.1102 (12) Å, α = 58.68 (8)°].

**Figure 2:**
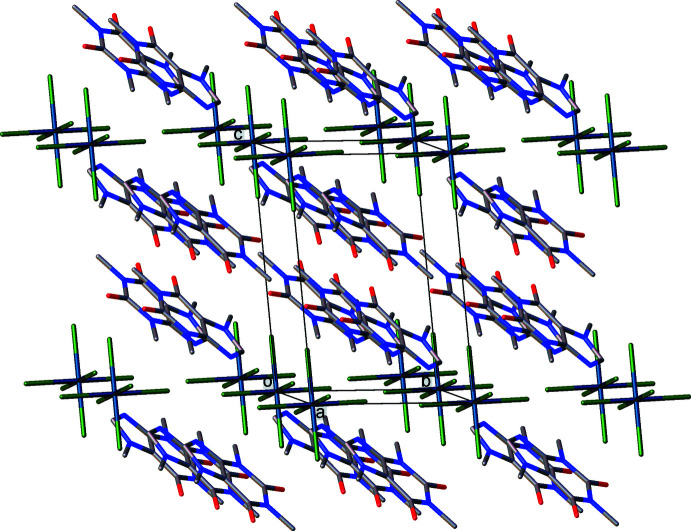
Crystal packing of **I**, showing the pseudo-layered character.

The crystal packing in **I** can be represented as cationic and anionic layers parallel to the (001) plane (Fig. 2 ▸). The caffeinium cations are linked by π-stacking inter­actions and weak C—H⋯O hydrogen bonds into double layers, which are connected to the anionic layers by hydrogen bonds of the N—H⋯Cl and C—H⋯C types (Table 2 ▸), the N1—H1⋯Cl3^ii^ [symmetry code: (ii) −*x* + 1, −*y* + 1, −*z*] inter­action being the strongest.

## 4. Hirshfeld surface analysis

**Figure 3:**
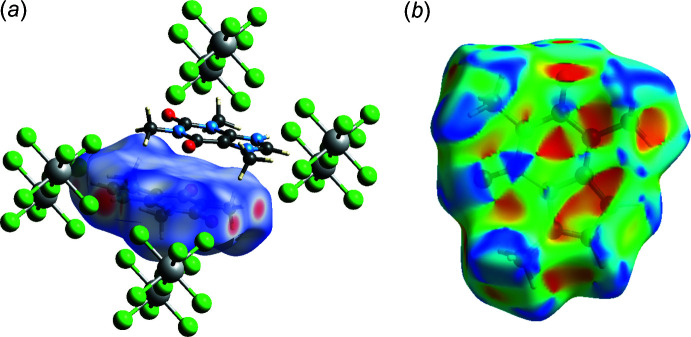
Hirshfeld surface of the caffeinium cation mapped over (*a*) *d*_norm_ and (*b*) shape-index.

*Crystal Explorer 21* was used to calculate the Hirshfeld surfaces (HS) and two-dimensional fingerprint plots (Figs. 3 ▸ and 4 ▸). The donor and acceptor groups are visualized using a standard (high) surface resolution and *d*_norm_ surfaces are mapped over a fixed colour scale of −0.401 (red) to 1.063 (blue) for cation and −0.402 to 0.934 a.u. for anion, as illus­trated in Fig. 3 ▸. Additionally, characteristic red and blue triangles indicative of π-stacking inter­actions are observed on the shape-index surface (Fig. 3 ▸ *b*).

**Figure 4:**
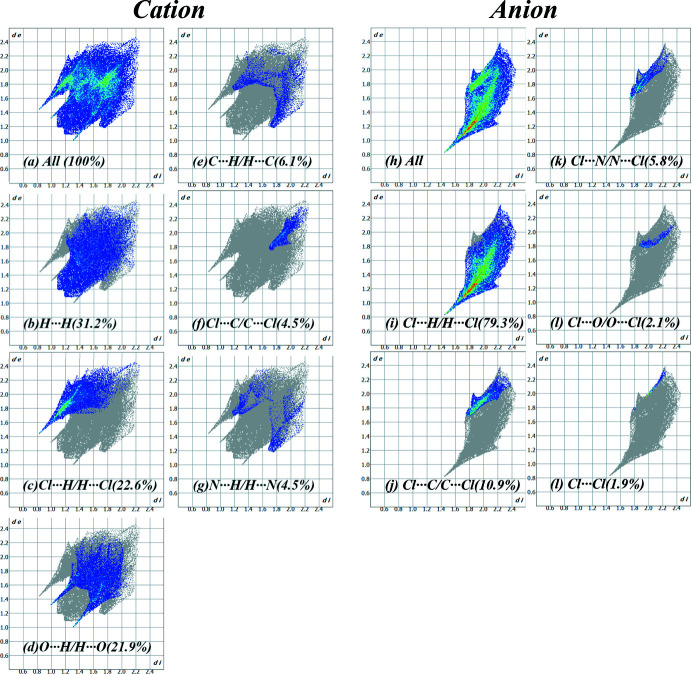
Two-dimensional fingerprint plots for the cations and anions in **I**.

**Figure 5:**
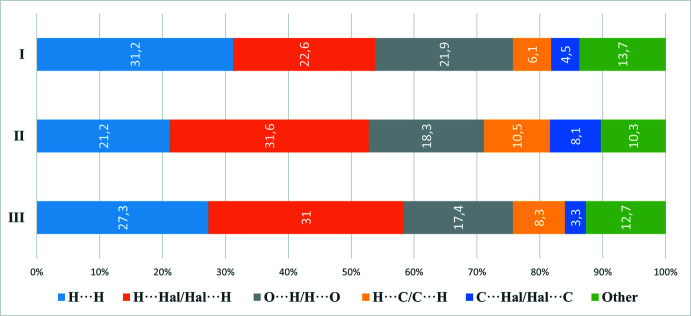
Percentage contributions of inter­molecular inter­actions for the cations in **I** and similar compounds.

**Figure 6:**
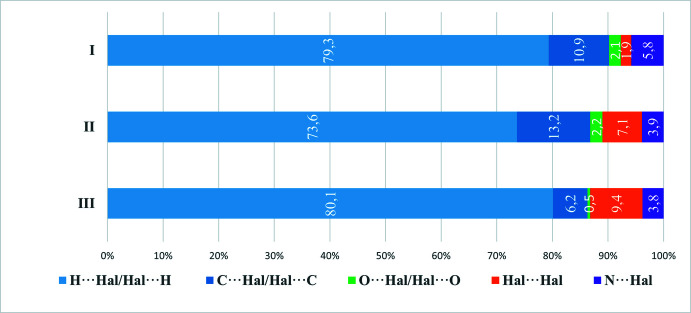
Percentage contributions of inter­molecular inter­actions for the anions in **I** and similar compounds.

Analysis of inter­molecular contacts shows that for the caffeinum cation, the largest contributions are made by H⋯H, Cl⋯H/H⋯Cl and O⋯H/H⋯O contacts (Fig. 5 ▸), and for the anion, by Cl⋯H/H⋯Cl and Cl⋯C/C⋯Cl contacts (Fig. 6 ▸). Whereas H⋯H contacts correspond to van der Waals inter­actions, O⋯H and Cl⋯H contacts can be described as weak hydrogen bonds. Typically, hydrogen bonds are revealed by characteristic discrete ‘spikes’ in the fingerprint plots – indeed, such features can be observed in Fig. 4 ▸ *c*,*d*,*i*. The structures of **II** and **III** show distributions of contacts (Figs. 5 ▸ and 6 ▸) broadly similar to that of **I**, if corrected for the different cation–anion ratios (1:1 for **I** and **III**, 2:1 for **II**).

## 5. Database survey

A search of the Cambridge Structural Database (CSD, Version 5.43, update of November 2022; Groom *et al.*, 2016 ▸) revealed 13 unique structures with caffeinium cations, but none of them contained anions of MHal_6_ type. The closest analogues of **I** found were **II** and **III** (see above), the former containing N-protonated 3-carb­oxy­pyridine (nicotinic acid) as the cation and [PtCl_6_]^2−^ as the anion, the latter containing a caffeinium cation with a methyl­ated (rather than protonated) N1 atom and a PF_6_^−^ anion.

## 6. Synthesis and crystallization

A saturated solution of dried caffeine in 5 mL of methanol was prepared, to which a few drops of a concentrated solution of hexa­chloro­platinic acid in hydro­chloric acid were added. After one week, the yellow crystals of **I** that formed were extracted from the solution.

## 7. Refinement

Crystal data, data collection and structure refinement details are summarized in Table 3 ▸. Reflections with resolution > 5 Å, obscured by the beamstop (beam diameter 0.6 mm), were excluded from the refinement. The methyl groups C5H_3_ and C7H_3_ were refined as rigid bodies rotating around N—C bonds [*U*_iso_(H) refined], C4H_3_ as rotationally disordered between two orientations with occupancies of 0.62 (4) and 0.38 (4) [*U*_iso_(H) = 1.2*U*_eq_(C)], with C—H 0.96 Å in each case. The H atoms at N1 and C3 were refined isotropically.

## Supplementary Material

Crystal structure: contains datablock(s) I. DOI: 10.1107/S2056989023005157/zv2027sup1.cif

Structure factors: contains datablock(s) I. DOI: 10.1107/S2056989023005157/zv2027Isup2.hkl

CCDC reference: 2268577

Additional supporting information:  crystallographic information; 3D view; checkCIF report

## Figures and Tables

**Table 1:** Selected geometric parameters (Å, °)

Pt1—Cl1	2.3153 (6)	Pt1—Cl3	2.3222 (6)
Pt1—Cl2	2.3161 (6)		
			
Cl1—Pt1—Cl2	89.97 (2)	Cl1^i^—Pt1—Cl3	89.93 (2)
Cl1^i^—Pt1—Cl2	90.03 (2)	Cl2^i^—Pt1—Cl3^i^	89.48 (3)
Cl1—Pt1—Cl3	90.07 (2)	Cl2—Pt1—Cl3^i^	90.52 (3)

Symmetry code: (i) 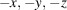 .

**Table 2:** Hydrogen-bond geometry (Å, °)

*D*—H⋯*A*	*D*—H	H⋯*A*	*D*⋯*A*	*D*—H⋯*A*
N1—H1⋯Cl3^ii^	0.85 (2)	2.45 (2)	3.296 (2)	174 (3)
C3—H3⋯Cl2^ii^	0.89 (3)	2.81 (3)	3.455 (3)	131 (3)
C5—H5*C*⋯Cl2	0.96	2.91	3.563 (3)	127
C7—H7*B*⋯O1^iii^	0.96	2.44	3.346 (4)	157

Symmetry codes: (ii) 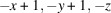 ; (iii) 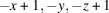 .

**Table 3:** Experimental details

Crystal data	
Chemical formula	(C_8_H_11_N_4_O_2_)_2_[PtCl_6_]
*M*_r_	798.20
Crystal system, space group	Triclinic, *P* 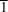
Temperature (K)	296
*a*, *b*, *c* (Å)	7.8800 (2), 8.1542 (2), 10.5374 (3)
α, β, γ (°)	95.784 (2), 91.525 (2), 112.472 (1)
*V* (Å^3^)	620.92 (3)
*Z*	1
Radiation type	Mo *K*α
μ (mm^−1^)	6.34
Crystal size (mm)	0.18 × 0.08 × 0.02

Data collection	
Diffractometer	Bruker Kappa APEXII area-detector
Absorption correction	Multi-scan (*SADABS*; Krause *et al.*, 2015 ▸)
*T*_min_, *T*_max_	0.734, 1.000
No. of measured, independent and observed [*I* > 2σ(*I*)] reflections	9764, 3616, 3573
*R*_int_	0.035
(sin θ/λ)_max_ (Å^−1^)	0.703

Refinement	
*R*[*F* ^2^ > 2σ(*F* ^2^)], *wR*(*F* ^2^), *S*	0.022, 0.041, 1.04
No. of reflections	3616
No. of parameters	173
No. of restraints	1
H-atom treatment	H atoms treated by a mixture of independent and constrained refinement
Δρ_max_, Δρ_min_ (e Å^−3^)	0.45, −0.89

Computer programs: *APEX3* (Bruker, 2018 ▸), *SAINT* (Bruker, 2013 ▸), *SHELXT2018/2* (Sheldrick, 2015*a* ▸), *SHELXL2018/3* (Sheldrick, 2015*b* ▸) and *OLEX2* (Dolomanov *et al.*, 2009 ▸).

## supplementary crystallographic information

### Crystal data

**Table:**

(C_8_H_11_N_4_O_2_)_2_[PtCl_6_]	*Z* = 1
*M**_r_* = 798.20	*F*(000) = 386
Triclinic, *P*1	*D*_x_ = 2.135 Mg m^−^^3^
*a* = 7.8800 (2) Å	Mo *K*α radiation, λ = 0.71073 Å
*b* = 8.1542 (2) Å	Cell parameters from 4212 reflections
*c* = 10.5374 (3) Å	θ = 3.1–29.8°
α = 95.784 (2)°	µ = 6.34 mm^−^^1^
β = 91.525 (2)°	*T* = 296 K
γ = 112.472 (1)°	Plate, orange
*V* = 620.92 (3) Å^3^	0.18 × 0.08 × 0.02 mm

### Data collection

**Table:**

Bruker Kappa APEXII area-detector diffractometer	3573 reflections with *I* > 2σ(*I*)
φ and ω scans	*R*_int_ = 0.035
Absorption correction: multi-scan (SADABS; Krause *et al.*, 2015)	θ_max_ = 30.0°, θ_min_ = 3.5°
*T*_min_ = 0.734, *T*_max_ = 1.000	*h* = −11→11
9764 measured reflections	*k* = −11→11
3616 independent reflections	*l* = −14→14

### Refinement

**Table:**

Refinement on *F*^2^	Primary atom site location: dual
Least-squares matrix: full	Hydrogen site location: mixed
*R*[*F*^2^ > 2σ(*F*^2^)] = 0.022	H atoms treated by a mixture of independent and constrained refinement
*wR*(*F*^2^) = 0.041	*w* = 1/[σ^2^(*F*_o_^2^) + (0.0171*P*)^2^] where *P* = (*F*_o_^2^ + 2*F*_c_^2^)/3
*S* = 1.04	(Δ/σ)_max_ < 0.001
3616 reflections	Δρ_max_ = 0.45 e Å^−^^3^
173 parameters	Δρ_min_ = −0.89 e Å^−^^3^
1 restraint	

### Special details

**Table:**

Geometry. All esds (except the esd in the dihedral angle between two l.s. planes) are estimated using the full covariance matrix. The cell esds are taken into account individually in the estimation of esds in distances, angles and torsion angles; correlations between esds in cell parameters are only used when they are defined by crystal symmetry. An approximate (isotropic) treatment of cell esds is used for estimating esds involving l.s. planes.

### Fractional atomic coordinates and isotropic or equivalent isotropic displacement parameters (Å^2^)

**Table:**

	*x*	*y*	*z*	*U*_iso_*/*U*_eq_	Occ. (<1)
Pt1	0.000000	0.000000	0.000000	0.02050 (4)	
Cl1	−0.04784 (10)	0.01026 (9)	0.21599 (5)	0.03560 (15)	
Cl2	0.30304 (9)	0.03978 (10)	0.04685 (6)	0.03557 (14)	
Cl3	0.08989 (10)	0.30803 (8)	0.01376 (6)	0.03532 (14)	
O1	0.5394 (3)	0.1187 (3)	0.3683 (2)	0.0474 (5)	
O2	0.1510 (3)	0.4010 (3)	0.4685 (2)	0.0517 (6)	
N1	0.5642 (3)	0.6187 (3)	0.1713 (2)	0.0340 (5)	
H1	0.656 (3)	0.646 (4)	0.126 (3)	0.053 (10)*	
N2	0.3378 (3)	0.6487 (3)	0.2696 (2)	0.0317 (5)	
N3	0.5758 (3)	0.3628 (3)	0.2700 (2)	0.0296 (5)	
N4	0.3502 (3)	0.2641 (3)	0.42037 (19)	0.0289 (5)	
C1	0.3648 (4)	0.5055 (3)	0.3126 (2)	0.0271 (5)	
C2	0.5073 (4)	0.4880 (3)	0.2510 (2)	0.0269 (5)	
C3	0.4589 (4)	0.7135 (4)	0.1859 (3)	0.0378 (7)	
H3	0.475 (4)	0.812 (4)	0.150 (3)	0.046 (9)*	
C4	0.1984 (5)	0.7171 (4)	0.3115 (3)	0.0485 (8)	
H4A	0.127241	0.645910	0.373253	0.058*	0.38 (4)
H4B	0.258237	0.838989	0.349513	0.058*	0.38 (4)
H4C	0.118735	0.711220	0.239191	0.058*	0.38 (4)
H4D	0.208901	0.818169	0.268052	0.058*	0.62 (4)
H4E	0.077905	0.625090	0.291792	0.058*	0.62 (4)
H4F	0.217407	0.752859	0.402113	0.058*	0.62 (4)
C5	0.7299 (4)	0.3460 (4)	0.2023 (3)	0.0461 (8)	
H5A	0.817041	0.334293	0.262454	0.097 (9)*	
H5B	0.788825	0.450488	0.160388	0.097 (9)*	
H5C	0.684455	0.242150	0.139850	0.097 (9)*	
C6	0.2746 (4)	0.3915 (3)	0.4059 (2)	0.0306 (5)	
C7	0.2650 (4)	0.1360 (4)	0.5105 (3)	0.0387 (7)	
H7A	0.226895	0.194389	0.581227	0.051 (5)*	
H7B	0.352395	0.090814	0.541266	0.051 (5)*	
H7C	0.159753	0.038818	0.468151	0.051 (5)*	
C8	0.4901 (4)	0.2398 (4)	0.3536 (2)	0.0312 (6)	

### Atomic displacement parameters (Å^2^)

**Table:**

	*U*^11^	*U*^22^	*U*^33^	*U*^12^	*U*^13^	*U*^23^
Pt1	0.02068 (7)	0.02260 (7)	0.01919 (6)	0.00823 (5)	0.00384 (4)	0.00678 (4)
Cl1	0.0415 (4)	0.0448 (4)	0.0214 (3)	0.0164 (3)	0.0078 (2)	0.0081 (2)
Cl2	0.0232 (3)	0.0458 (4)	0.0396 (3)	0.0136 (3)	0.0015 (2)	0.0125 (3)
Cl3	0.0420 (4)	0.0236 (3)	0.0410 (3)	0.0114 (3)	0.0123 (3)	0.0088 (2)
O1	0.0488 (13)	0.0419 (12)	0.0622 (14)	0.0242 (11)	0.0122 (10)	0.0258 (10)
O2	0.0584 (15)	0.0519 (14)	0.0569 (13)	0.0294 (12)	0.0344 (11)	0.0201 (11)
N1	0.0342 (13)	0.0317 (12)	0.0351 (12)	0.0088 (10)	0.0109 (10)	0.0131 (9)
N2	0.0337 (12)	0.0253 (11)	0.0357 (11)	0.0106 (10)	0.0019 (9)	0.0054 (9)
N3	0.0285 (12)	0.0289 (11)	0.0327 (11)	0.0110 (9)	0.0066 (9)	0.0085 (8)
N4	0.0331 (12)	0.0278 (11)	0.0258 (10)	0.0103 (9)	0.0052 (8)	0.0090 (8)
C1	0.0280 (13)	0.0218 (12)	0.0303 (12)	0.0079 (10)	0.0015 (10)	0.0040 (9)
C2	0.0288 (13)	0.0236 (12)	0.0251 (11)	0.0062 (10)	0.0009 (9)	0.0049 (9)
C3	0.0453 (18)	0.0281 (14)	0.0393 (15)	0.0110 (13)	0.0048 (12)	0.0129 (11)
C4	0.0430 (18)	0.0396 (18)	0.071 (2)	0.0243 (15)	0.0078 (16)	0.0092 (15)
C5	0.0368 (17)	0.0457 (18)	0.062 (2)	0.0201 (14)	0.0206 (14)	0.0134 (15)
C6	0.0328 (14)	0.0288 (13)	0.0283 (12)	0.0097 (11)	0.0043 (10)	0.0033 (10)
C7	0.0433 (17)	0.0378 (16)	0.0320 (13)	0.0087 (13)	0.0098 (12)	0.0172 (11)
C8	0.0290 (14)	0.0296 (14)	0.0329 (13)	0.0081 (11)	0.0001 (10)	0.0082 (10)

### Geometric parameters (Å, º)

**Table:**

Pt1—Cl1^i^	2.3153 (6)	N4—C7	1.464 (3)
Pt1—Cl1	2.3153 (6)	N4—C8	1.389 (3)
Pt1—Cl2^i^	2.3161 (6)	C1—C2	1.358 (4)
Pt1—Cl2	2.3161 (6)	C1—C6	1.433 (3)
Pt1—Cl3	2.3222 (6)	C3—H3	0.89 (3)
Pt1—Cl3^i^	2.3222 (6)	C4—H4A	0.9600
O1—C8	1.213 (3)	C4—H4B	0.9600
O2—C6	1.213 (3)	C4—H4C	0.9600
N1—H1	0.846 (18)	C4—H4D	0.9600
N1—C2	1.370 (3)	C4—H4E	0.9600
N1—C3	1.335 (4)	C4—H4F	0.9600
N2—C1	1.380 (3)	C5—H5A	0.9600
N2—C3	1.312 (4)	C5—H5B	0.9600
N2—C4	1.469 (4)	C5—H5C	0.9600
N3—C2	1.354 (3)	C7—H7A	0.9600
N3—C5	1.468 (3)	C7—H7B	0.9600
N3—C8	1.389 (3)	C7—H7C	0.9600
N4—C6	1.399 (3)		
			
Cl1^i^—Pt1—Cl1	180.0	C1—C2—N1	107.3 (2)
Cl1—Pt1—Cl2	89.97 (2)	N1—C3—H3	125 (2)
Cl1—Pt1—Cl2^i^	90.03 (2)	N2—C3—N1	109.9 (2)
Cl1^i^—Pt1—Cl2	90.03 (2)	N2—C3—H3	125 (2)
Cl1^i^—Pt1—Cl2^i^	89.97 (2)	N2—C4—H4A	109.5
Cl1—Pt1—Cl3^i^	89.93 (2)	N2—C4—H4B	109.5
Cl1—Pt1—Cl3	90.07 (2)	N2—C4—H4C	109.5
Cl1^i^—Pt1—Cl3	89.93 (2)	H4A—C4—H4B	109.5
Cl1^i^—Pt1—Cl3^i^	90.07 (2)	H4A—C4—H4C	109.5
Cl2—Pt1—Cl2^i^	180.0	H4B—C4—H4C	109.5
Cl2^i^—Pt1—Cl3	90.52 (3)	H4D—C4—H4E	109.5
Cl2—Pt1—Cl3	89.48 (3)	H4D—C4—H4F	109.5
Cl2^i^—Pt1—Cl3^i^	89.48 (3)	H4E—C4—H4F	109.5
Cl2—Pt1—Cl3^i^	90.52 (3)	N3—C5—H5A	109.5
Cl3^i^—Pt1—Cl3	180.0	N3—C5—H5B	109.5
C2—N1—H1	128 (2)	N3—C5—H5C	109.5
C3—N1—H1	124 (2)	H5A—C5—H5B	109.5
C3—N1—C2	107.7 (2)	H5A—C5—H5C	109.5
C1—N2—C4	125.7 (2)	H5B—C5—H5C	109.5
C3—N2—C1	108.2 (2)	O2—C6—N4	122.2 (2)
C3—N2—C4	126.1 (2)	O2—C6—C1	126.5 (3)
C2—N3—C5	123.2 (2)	N4—C6—C1	111.2 (2)
C2—N3—C8	117.9 (2)	N4—C7—H7A	109.5
C8—N3—C5	118.8 (2)	N4—C7—H7B	109.5
C6—N4—C7	116.2 (2)	N4—C7—H7C	109.5
C8—N4—C6	127.2 (2)	H7A—C7—H7B	109.5
C8—N4—C7	116.5 (2)	H7A—C7—H7C	109.5
N2—C1—C6	131.1 (2)	H7B—C7—H7C	109.5
C2—C1—N2	106.9 (2)	O1—C8—N3	120.5 (3)
C2—C1—C6	122.0 (2)	O1—C8—N4	122.1 (2)
N3—C2—N1	128.6 (2)	N4—C8—N3	117.3 (2)
N3—C2—C1	124.1 (2)		
			
N2—C1—C2—N1	−0.4 (3)	C5—N3—C2—N1	−0.8 (4)
N2—C1—C2—N3	179.0 (2)	C5—N3—C2—C1	179.9 (3)
N2—C1—C6—O2	−0.4 (5)	C5—N3—C8—O1	−1.5 (4)
N2—C1—C6—N4	−179.0 (2)	C5—N3—C8—N4	177.2 (2)
C1—N2—C3—N1	0.3 (3)	C6—N4—C8—O1	−175.8 (3)
C2—N1—C3—N2	−0.6 (3)	C6—N4—C8—N3	5.4 (4)
C2—N3—C8—O1	175.7 (2)	C6—C1—C2—N1	−178.6 (2)
C2—N3—C8—N4	−5.5 (3)	C6—C1—C2—N3	0.7 (4)
C2—C1—C6—O2	177.4 (3)	C7—N4—C6—O2	3.3 (4)
C2—C1—C6—N4	−1.2 (3)	C7—N4—C6—C1	−178.1 (2)
C3—N1—C2—N3	−178.7 (3)	C7—N4—C8—O1	0.3 (4)
C3—N1—C2—C1	0.6 (3)	C7—N4—C8—N3	−178.4 (2)
C3—N2—C1—C2	0.0 (3)	C8—N3—C2—N1	−178.0 (3)
C3—N2—C1—C6	178.1 (3)	C8—N3—C2—C1	2.8 (4)
C4—N2—C1—C2	−179.4 (3)	C8—N4—C6—O2	179.4 (3)
C4—N2—C1—C6	−1.4 (4)	C8—N4—C6—C1	−2.0 (4)
C4—N2—C3—N1	179.8 (3)		

Symmetry code: (i) −*x*, −*y*, −*z*.

### Hydrogen-bond geometry (Å, º)

**Table:**

*D*—H···*A*	*D*—H	H···*A*	*D*···*A*	*D*—H···*A*
N1—H1···Cl3^ii^	0.85 (2)	2.45 (2)	3.296 (2)	174 (3)
C3—H3···Cl2^ii^	0.89 (3)	2.81 (3)	3.455 (3)	131 (3)
C5—H5*C*···Cl2	0.96	2.91	3.563 (3)	127
C7—H7*B*···O1^iii^	0.96	2.44	3.346 (4)	157

Symmetry codes: (ii) −*x*+1, −*y*+1, −*z*; (iii) −*x*+1, −*y*, −*z*+1.
